# Contrasting emergence of Lyme disease across ecosystems

**DOI:** 10.1038/ncomms11882

**Published:** 2016-06-16

**Authors:** Atle Mysterud, William Ryan Easterday, Vetle Malmer Stigum, Anders Bjørnsgaard Aas, Erling L. Meisingset, Hildegunn Viljugrein

**Affiliations:** 1 Centre for Ecological and Evolutionary Synthesis (CEES), Department of Biosciences, University of Oslo, PO Box 1066 Blindern, NO-0316 Oslo, Norway; 2 Section for Genetics and Evolutionary Biology (EVOGENE), Department of Biosciences, University of Oslo, PO Box 1066 Blindern, NO-0316 Oslo, Norway; 3 Norwegian Institute of Bioeconomy Research, Division of Forest and Forest Resources, Tingvoll Gard, NO-6630 Tingvoll, Norway; 4 Norwegian Veterinary Institute, PO Box 750 Sentrum, NO-0106 Oslo, Norway

## Abstract

Global environmental changes are causing Lyme disease to emerge in Europe. The life cycle of *Ixodes ricinus*, the tick vector of Lyme disease, involves an ontogenetic niche shift, from the larval and nymphal stages utilizing a wide range of hosts, picking up the pathogens causing Lyme disease from small vertebrates, to the adult stage depending on larger (non-transmission) hosts, typically deer. Because of this complexity the role of different host species for emergence of Lyme disease remains controversial. Here, by analysing long-term data on incidence in humans over a broad geographical scale in Norway, we show that both high spatial and temporal deer population density increase Lyme disease incidence. However, the trajectories of deer population sizes play an overall limited role for the recent emergence of the disease. Our study suggests that managing deer populations will have some effect on disease incidence, but that Lyme disease may nevertheless increase as multiple drivers are involved.

## Abstract (web summary)

Environmental change is thought to have driven the recent emergence of Lyme disease in Europe. Using a decade of human disease incidence data across a large area in Norway, Mysterud *et al.* show that incidence correlates with deer population, but that deer population plays a limited role in recent disease emergence.

## Graphical abstract

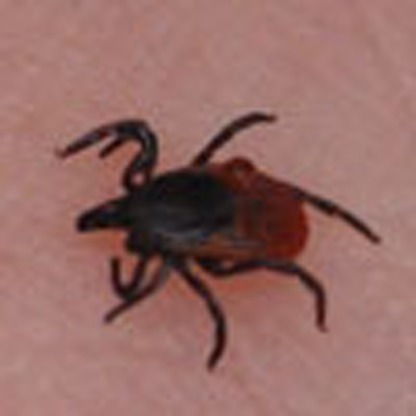

Global environmental changes affect several drivers of emergence of vector-borne diseases that are typically difficult to unravel in terms of their role in complex epidemiological networks^1^,^2^. The blacklegged tick *Ixodes scapularis* in North America and the sheep tick *Ixodes ricinus* in Europe are the most important vectors of spirochetes within the *Borrelia burgdorferi* sensu lato (s.l.) complex, the causal agent of Lyme disease (LD)^3^,^4^, and of several other pathogens causing human and animal diseases^5^. Climate warming and land use change are contributing to the tick distribution to expand upwards in elevation and towards higher latitudes in Northern Europe^6^. The hazard of LD for humans is linked to both vector abundance and infection prevalence^7^. These factors are determined by the abundance and composition of different-sized vertebrate hosts suitable for different life stages of ticks and whether they are competent reservoirs of the pathogen^8^,^9^.

The life cycle of these ticks involves three stages, each require a blood meal to moult into the next stage or reproduce^10^. Larvae and nymphs parasitize a wide range of different sized vertebrate hosts, picking up the pathogen from small vertebrates, while adult female ticks require a meal from a large vertebrate (>1 kg) for reproduction. Hence, the ontogenetic niche shift leads to a complicated tick population regulation that depends on the availability of both large (critical for adults) and small (used by larvae) hosts^10^,^11^. In North America, access to white-tailed deer *Odocoileus virginianus* or other large hosts are required for tick reproduction, but annual fluctuations in deer numbers play a minor role in limiting LD under conditions of moderate to high deer density^12^,^13^. The red deer *Cervus elaphus* and roe deer *Capreolus capreolus* have markedly expanded over recent decades and are the most widely distributed large hosts available for ticks in Europe^14^. There is evidence that deer in Europe amplify tick populations^15^, but the net effect on disease risk and incidence remains to be established.

The ratio of competent to incompetent transmission hosts is central for the prevalence of pathogens in the tick population. In the United States, the main reservoirs of *B. burgdorferi* s.l. is the white-footed mouse *Peromyscus leucopus*, eastern chipmunk *Tamias striatus* and shrews with a wide distribution^16^. Hence, with only three main transmission hosts, any additional host species in the community may lead to a higher proportion of larvae feeding on non-transmission hosts, lowering pathogen prevalence in the tick population. This is termed a dilution effect or buffering of disease with increasing biodiversity. Deer are regarded as incompetent transmission hosts, but must feed a sufficiently large amount of tick larvae to cause dilution of the pathogen in the vector population to be termed a dilution host^9^. In the United States, LD has been central to the biodiversity buffers disease paradigm^17^. The effectiveness of biodiversity diluting LD and other diseases has been heavily debated^17^,^18^,^19^. The buffering effect of biodiversity for LD has not been tested outside the United States. The LD systems differ across continents in terms of hosts, vectors and pathogens. In Europe, there are several pathogenic genospecies of *B. burgdorferi* s.l. Most human cases in Europe are caused by *Borrelia afzelii* having a small mammal reservoir and *Borrelia garinii* having a bird reservoir, while *B. burgdorferi* sensu stricto causing LD in North America is present, but less common. The larger base of reservoir hosts including both small mammals and birds in Europe^20^ suggests that dilution due to biodiversity is less likely^8^, and that rather the relative densities of transmission to non-transmission hosts will determine hazard. However, long-term and spatially extensive empirical data have been lacking from Northern Europe where LD is emerging.

We here analyse long-term data (1991–2012) on LD incidence in humans over a broad geographical scale (416 municipalities in Norway; latitudinal range 57° 58′ N–71° 08′ N) at the northern limits covering areas with recently emerging LD. This is made possible since systemic LD has status as a notifiable disease in Norway. We first tested if LD was emerging (an increase in incidence over time) and whether there was regional variation in level of emergence. An important aspect of this study is its geographical coverage because it includes regions with contrasting trajectories of population sizes of deer caused, in part, by different harvesting management regimes. These regions differ sufficiently to classify them as different ecosystems (see Methods). Hence, we relate the incidence of LD to the spatial contrasts and temporal variation in deer population density across and within four regions. In particular, we highlight the contrast between region West and South, with a steep increase and a decline in deer numbers over the monitoring period, respectively. We hypothesized that LD incidence may increase with higher deer population density in (P_1a_) space and (P_1b_) time, as deer are important tick reproduction hosts (LD-deer limitation hypothesis or H_1_ below.) We further tested whether an increasing deer density caused increased LD incidence over time, that is, an emergence, together with other drivers (P_1c_) or whether an increasing deer density was sufficiently strong to (P_1d_) fully explain the emergence across ecosystems. Finally, we tested whether biodiversity buffered LD incidence, predicting (P_2_) a lower LD incidence in areas with higher mammalian host species richness (biodiversity buffers disease hypothesis or H_2_ below). We statistically took into account the impact of climate, land use, a proxy for rodent abundances, and a proxy for human connectedness to the landscape. Finally, in the West region, which has experienced a marked increase in red deer, we used experimental data (exclosures), spatially extensive flagging data for questing (that is, host seeking) sheep tick abundance, tick load on red deer ears and pathogen prevalence to document the mechanisms by which red deer density might affect LD incidence. We find that both high spatial and temporal deer population densities increase LD incidence, but that the trajectories of deer population sizes could not fully explain the recent emergence of LD. We conclude that managing deer populations will have some effect on LD incidence, but that such actions are not necessarily sufficient to hinder emergence as multiple drivers are involved.

## Results

### Analysis of LD incidence

**Figure 1:**
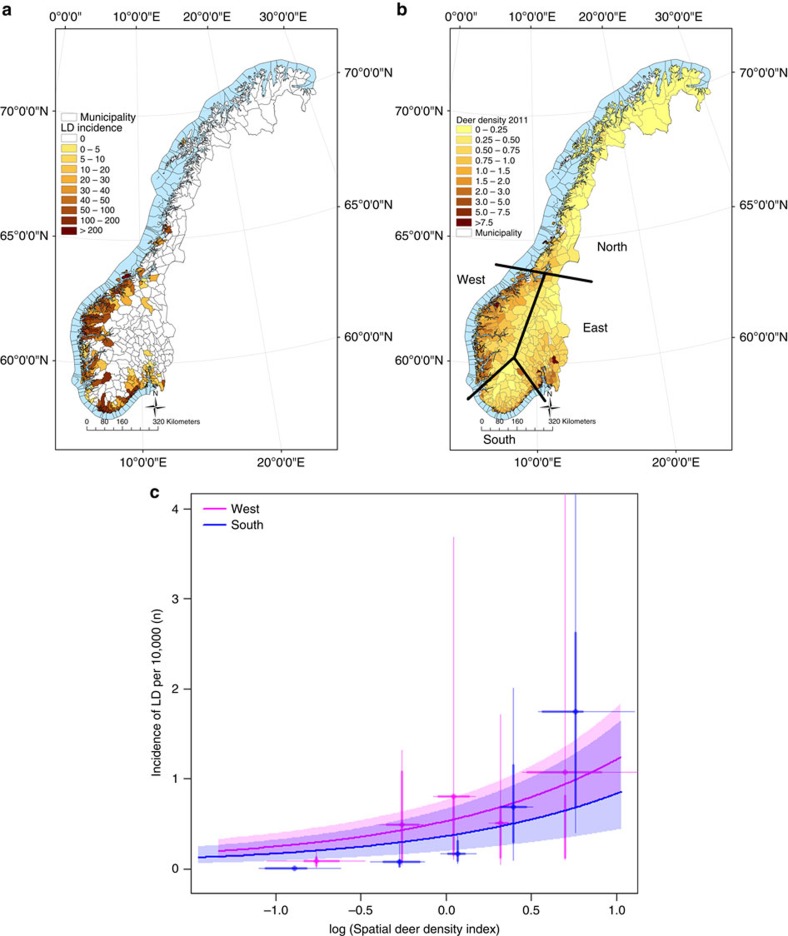
Spatial variation in Lyme disease incidence and deer population index in Norway. (**a**) Incidences of Lyme disease in humans are pooled over the 5-year period 2008–2012 (per 100,000). (**b**) The deer population index (number of harvested deer per km^2^) for all municipalities of Norway is given for year 2011. Numbers of deer per year are pooled for all deer species (roe deer, red deer and moose). (**c**) The relationship between LD incidence (per 10,000) and the (log) spatial population density index of deer plotted for year 2011 for regions West and South in Norway. Analysis includes 2,007 LD cases from 416 municipalities. Points are averages of residuals for binned ranges of data; thin and thick lines are 80% and 50% of the data, respectively. See Methods for classification of regions.

**Figure 2:**
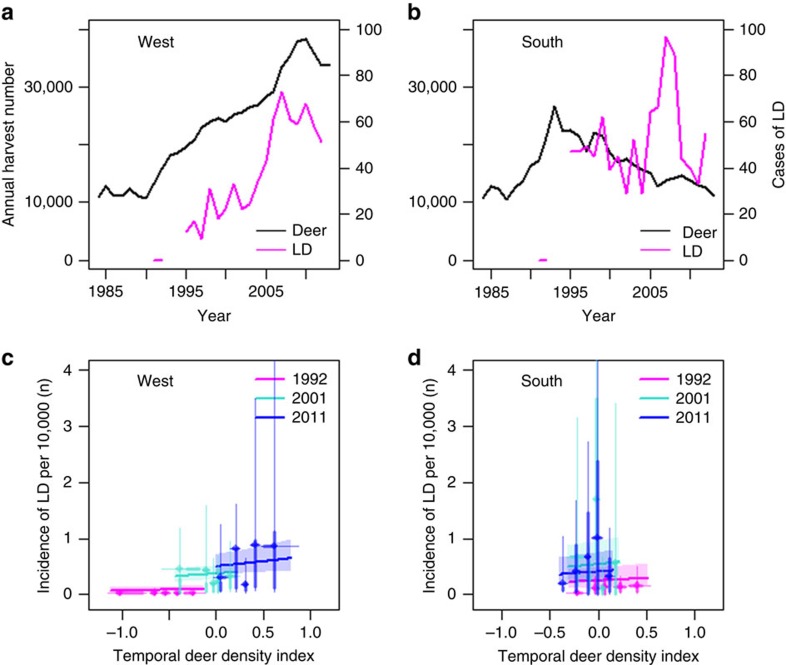
Temporal variation in Lyme disease incidence and deer population index in Norway. The annual number of harvested deer and Lyme disease cases in humans in region (**a**) West and (**b**) South and the relationship between LD incidence (per 10,000) and the temporal variation in deer density index in the (**c**) West and (**d**) South regions of Norway for years 1991, 2001 and 2011. Analysis includes 2,007 LD cases from 416 municipalities. Points are averages of residuals for binned ranges of data; thin and thick lines are 80% and 50% of the data, respectively. Numbers of deer per year are pooled for all deer species (roe deer, red deer and moose).

The incidence of LD was clearly emerging, but with significant differences in yearly increases across the four regions of Norway (Table 1). LD incidence was related to both the spatial population density of all deer species combined (referred to as deer, P_1a_, Fig. 1) and the temporal component (annual residual relative to mean density on a log scale) of the deer population (P_1b_, Fig. 2), providing support for two of the predictions from the LD-deer limitation hypothesis. First, the temporal increase of the deer population size was correlated with the emergence of LD in the region West (Fig. 2c, partial support of P_1c_, see Methods for the working definition of West and all other regional divisions), which has shown a marked density increase in red deer in recent decades (Fig. 2a, Supplementary Table 1 and Supplementary Note 1). In contrast, areas along the South coast and towards the East, which are dominated by roe deer and moose (*Alces alces*), have seen a decline in their numbers (Fig. 2b). Here incidence was not linked to temporal population density trajectory of all deer species combined (Fig. 2d). However, even for the West region, only a small proportion of the LD emergence could be attributed to the red deer density increase (Fig. 2c; rejecting P_1d_), suggesting that other drivers were involved and important for emergence. There was no evidence that LD was buffered by biodiversity measured as mammal species richness (rejecting H_2_; Table 2). Regions East and North have much lower LD incidence compared with West and South (Table 1), the latter two regions thus providing our main comparison of how deer density correlates with patterns of LD emergence.

The incidence of LD was correlated with proxies of spatial (the distance to the coast) and temporal variation in climate (annual variation in the North Atlantic Oscillation, Table 1). Controlling for the distance to the coast was important as ticks thrive along the coastline, but this variable is also correlated to a higher spatial density of deer (particularly in region South; Supplementary Table 2). The spatial contrast in deer density had a mainly positive effect on the incidence of LD while accounting for distance to the coast (Table 1). However, the effect of spatial deer density is slightly higher if distance from the coast is removed from the model (estimated effect 0.711 versus 1.021). Similarly, covariation may cause the yearly trend to capture part of the temporal deer density effect, particularly in the region West. Hence, if running a model using a common year trend for all regions (that is, excluding ‘year × region'), the estimated effect of temporal deer density increases (estimated effect 0.085 versus 0.178). Thus, our estimates of the effect of spatial and temporal deer density effects are conservative. Some variables of land use were also important (Table 1), but we failed to link LD to a proxy of rodent abundances, as adding such a proxy did not improve explanatory power of the model based on Akaike information criterion (AIC; Table 2).

We cannot exclude that the increased awareness of LD is leading to higher rates of reporting over time and hence play a role in the emergence pattern. Increased reporting might play a role in the first few years, but rerunning models from 1995 onwards gave qualitatively similar results. Also, the emergence of babesiosis^21^, anaplasmosis and tick-borne encephalitis^22^ suggests the existence of some common environmental driver, and clearly the spatial effect of deer density and temporal effect of deer density after controlling for year trends cannot be explained by a potential reporting bias. In the United States, the true LD incidence was 10-fold higher than the official number of reported cases^23^. As a result of our statistical data only include systemic LD infections, we are likely only seeing the tip of the iceberg in terms of LD incidence.

### Tick population and deer density in region West

**Figure 3:**
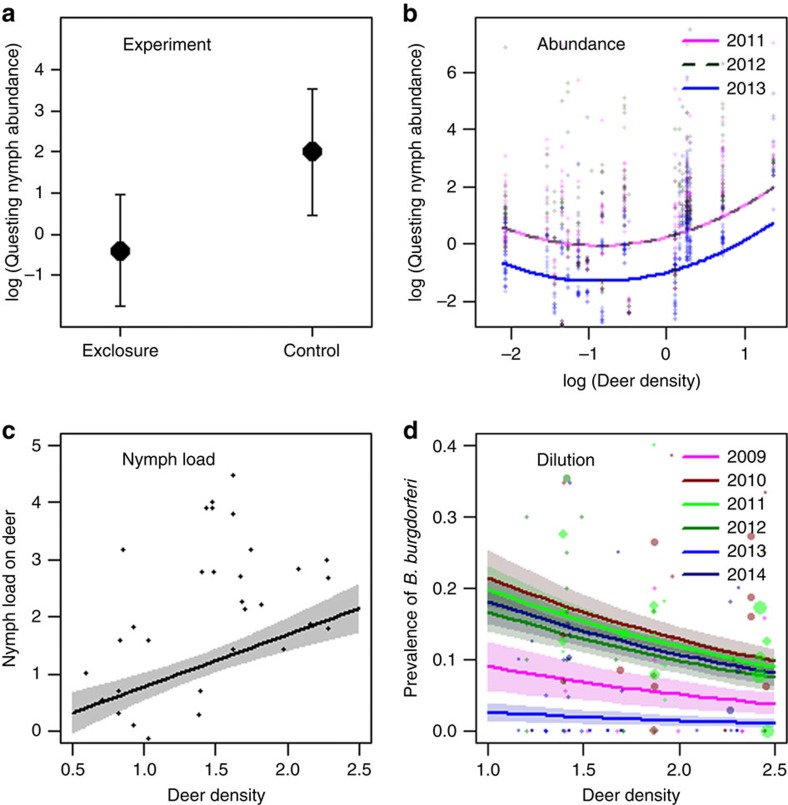
Linking tick abundances and pathogen dilution to deer density. Relationship between (**a**) the (log) abundance of questing tick nymphs inside and outside of deer exclosures (10 replicates), (**b**) the (log) abundance of questing tick nymphs (*n*=11,216) and deer population density index, (**c**) the (log) nymphal tick load on red deer ears (*n*=49) and deer population density index, and (**d**) the prevalence of *Borrelia burgdorferi* sensu lato in ticks (*n*=3,324) and deer population density index along the west coast of Norway. For **d**, data points are proportional to (square root) sample size.

The red deer populations in the West region of Norway have rapidly increased in the last decades (Fig. 2a and Supplementary Table 1). We focused on determining the mechanisms behind the correlation between deer density and LD incidence in the two largest counties (Sogn and Fjordane, and Møre and Romsdal). The exclosure experiment showed that the complete eradication of deer resulted in significantly fewer questing nymphal ticks 3 years after the fences were erected (estimated mean density outside was 7.5 ticks versus 0.7 ticks per 20 m^2^ inside exclosures, Fig. 3a, *Z*=4.66, *P*<0.001). These results indicate that hosts for adult females limit the tick populations where large vertebrates are totally absent. The abundance of questing ticks was also linked to spatial contrasts of red deer density mainly caused by management in the West region of Norway (*n*=23,298 nymphal ticks from 71 transects). At the finer scale of local management units (*n*=21, mean size=103.6 km^2^) in Møre and Romsdal County the abundance of questing ticks was positively related to the red deer density in spring (Fig. 3b and Table 3). While at the broader scale of municipality (*n*=9, mean size=305.2 km^2^ removing mountain areas) in Sogn and Fjordane County (Table 3), where the contrasts in red deer density was about half of Møre and Romsdal County, the effect was weak and insignificant. There were more questing ticks in May than in August, and the abundance varied from year to year. The positive effect of red deer density in Møre and Romsdal County was robust when controlling for other environmental factors such as distance to the coast and slope (Table 3). However, distance to the coast and density of red deer is partially correlated; hence, removing distance to the coast as a covariate in the analysis amplified the effect of red deer density even further. Tick load (nymphs) on GPS-marked red deer (*n*=49) also increased considerably at high red deer densities (Fig. 3c and Table 5).

Our last objective was to estimate the effect of red deer density on the pathogen prevalence in nymphal ticks at different temporal scales (*n*=4,557; Table 6). During the main questing period in the spring (May), there was a reduction in pathogen (*B. burgdorferi* s.l.) prevalence with increasing red deer density (spatial contrast) in both counties. However, the effect was fairly weak (Fig. 3d, Supplementary Fig. 1 and Table 6). In contrast, there was no measurable effect of red deer density on pathogen prevalence in the late questing period (August) at this high latitude (Table 6). The prevalence of the pathogen varied largely among years. On the basis of 6 years of data, we were able to link the high pathogen prevalence in nymphs to the high abundances of rodents the previous year in Sogn and Fjordane County, but only in the month of May (Table 6). Analytically combining functions on tick amplification (Tables 4 and 5) and pathogen prevalence (Table 6) to assess number of infected nymphs show that the estimated functions were quite variable and model dependent, thus the overall number of infected nymphs was weakly related or unrelated to red deer density depending on season and year.

## Discussion

The main aim of this study was to test whether LD incidences at the northernmost distribution range in Europe have increased over time in recent decades (an emergence), whether patterns of increase differed across ecosystems, and the extent to which emergence could be linked to (H_1_) altered abundances of deer.

Reduced abundances of questing ticks have been reported when comparing data from inside and outside deer exclosures in a number of cases^15^,^24^,^25^ or when completely removing deer from islands and thereby rendering ticks with no alternative large hosts^25^. This outcome is not surprising because the tick population would then be strongly limited by the absence of reproduction hosts for adult females^12^. However, for mainland populations, it is not feasible and often not desired to remove deer completely and there may be other large hosts present. Our study shows that reducing deer densities within the range currently observed in mainland areas would only marginally affect the incidence of LD. In the region South of Norway, temporal variation in deer densities could not explain LD emergence (Fig. 2b,d). In contrast, in the region West of Norway, increased red deer density played a role for LD emergence (Fig. 2a,c), and we could mechanistically link red deer density to tick amplification through both tick questing abundances and tick load on red deer (Fig. 3). However, temporal trajectories of deer population sizes could not explain all of the increase of LD over time, even in the West region (Fig. 2c), suggesting that another driver is important across the whole region, most likely a warmer climate. The increase in white-tailed deer in the United States was in early phases of recolonization and population growth regarded as important for LD emergence^26^,^27^, while more recent studies find no relationship of LD risk^12^ or incidence^13^ unless very low deer densities are included in analyses. Our review of studies linking tick abundance to actual deer densities provides further support for a stronger effect of deer when going from low to medium compared with from medium to high deer population density (Supplementary Table 3). Indeed, a common feature of studies reporting a positive link between tick abundance and deer density is that they include a wide range of densities from well below 10 deer per km^2^ to quite high (Supplementary Table 3). Whether increased density of ticks with increased deer density leads to increased incidence of LD also depends on pathogen levels and exposure. The emergence of LD across contrasting temporal trajectories of deer density in Norway provides strong evidence that the emergence across northern Europe cannot be attributed to deer populations alone. Yet, deer density plays some role when comparing a wide range of densities including from low to high densities, still the effect of deer density is a weak predictor of LD incidence.

Unravelling the relationship between temporal trajectories of deer populations and the incidence of LD is difficult for several reasons. Time delays may be caused by both the tick life cycle, strong historical momentum in deer population dynamics driven by age structure^28^, and by short-term lags in the quota system when using harvest data as an index of density^29^. We solved this by calculating a spatial deer population density and a temporal deer population density index. Both an increase in the spatial (Fig. 1) and the temporal (Fig. 2) component of deer density was correlated with an increased LD incidence. This relationship held while retaining distance from the coast (a proxy for spatial variation in local climate) and ‘year' as a region-specific trend; thus, estimates of the deer density effect are both conservative and robust. This is in contrast to studies from the United States where annual variation in white-tailed deer (*O. virginianus*) density could not explain the variation in tick abundance and hazard (number of infected nymphs) over time. This lack of an effect from deer numbers in the United States was attributed to the presence of other medium-sized mammals when the deer density was reduced^12^. The strong negative effect on tick abundances by the eradication of deer from islands in the United States is thought to arise from the lack of alternative medium-sized hosts.

Rodents are the main transmission host for *B. burgdorferi* s.l. in the United States, and high number of infected nymphs in endemic areas of the United States has been linked to rodent population peaks^12^. In endemic areas of the United States, a temporal increase in LD incidence was suggested linked to increased abundance of rodents over time rather than to deer density^13^. Evidence for increasing rodent abundances was based on statistics of declining red fox (*Vulpes vulpes*) numbers, a small rodent predator, due to expansion of coyote (*Canis latrans*) populations that predate on red fox, that is, a trophic cascading effect. This increase in rodent populations contrasts with the trend in Europe, where climate change has caused a continental wide dampening of population cycles and declining abundances of rodents^30^. Further, for Scandinavia, red fox was almost eradicated during the 1980s due to a sarcoptic mange epidemic, but populations have been on the rebound from 1990 onwards^31^. Thus, there has been a reduction rather than an increase of rodent numbers coinciding with LD emergence at the northern ranges in Europe. We found no link between rodent cycles and annual variation in LD incidence in Norway. However, there was annual variation in pathogen prevalence linked to the rodent population cycle (Table 6), suggesting some role of rodents driving the LD hazard. The weak role of rodents for LD incidence may be due to a wider range of transmission hosts for *B. burgdorferi* s.l. in Europe, including also birds. The genospecies *B. afzelii* (61.6% (ref. 32); 68.4% (ref. 33)) were dominant and *B. garinii* were less prevalent (23.4% (ref. 32); 20.8% (ref. 33)) in studies of questing ticks in Norway. This suggests that small mammals are more important reservoir hosts than birds. Yet, the additional reservoir in birds may dampen the observed variation between rodent cycles and LD incidence.

The incidence of LD in North America has been important for the development of the biodiversity buffering disease paradigm^9^,^34^ (H_2_). Detailed ecological studies have found correlations between LD risk and both host community composition^35^ and host diversity^9^,^23^,^36^. Although this paradigm is widely supported across disease systems^19^, it is not universal^18^. In our study we found no effect of mammal species richness on LD incidence, as predicted by H_2_. Although our analysis encompasses a wide range of latitudes, the mammal communities only differed by a few species (10–17) within LD areas, and this structure was apparently not sufficient to cause any marked dilution effect. Whether a wider range of mammalian species richness would change this outcome is uncertain. White-footed mouse, eastern chipmunk and shrews are the main pathogen reservoirs in North America^12^,^16^, while in Europe, there are several more host-specific genospecies of *B. burgdorferi* s.l. and a wider range of pathogen reservoir hosts^20^. Of the 18 tick host species included in our analysis, as many as 10 are considered pathogen reservoir hosts^20^. Therefore, increased biodiversity in Europe may not necessarily contribute to a dilution effect as it does in North America^8^. Vertebrate communities in North America and Europe are also markedly different in other respects. In the United States, a recent broad analysis showed that states with high mammalian host richness (34 species) had reduced LD incidence^23^. In contrast, the mammalian host richness is much lower in the northern areas of Norway, only 18 mammalian tick hosts listed (Supplementary Note 2). North American ecosystems harbour a more diverse mammalian community, particularly within the medium-sized range. This rich community provides more alternative hosts to adult ticks than in northern Europe.

Nymphs are the most important stage for transmitting disease to humans, and the hazard to humans is linked to the density of infected nymphs^7^. Deer are non-transmission hosts^37^, and empirical evidence showed that reducing deer density on an island increased infection rates of nymphs^25^. The idea that deer populations can wash out or dilute the pathogen and reduce the LD risk to humans has nevertheless been heavily criticized in essays^17^,^18^. A dilution effect would require not only that deer feed larvae, which is the case for both roe deer^38^ and red deer^39^, but also that numbers are quantitatively high enough compared with the number of larvae fed by rodents and birds in a given area. We found that the relationship between prevalence of *B. burgdorferi* s.l. in ticks and deer population density varied seasonally at the same location. Our large-scale empirical results showed that a certain pathogen dilution caused by deer density was evident in the main questing period. Furthermore, there was a lack of dilution effect in the fall, which suggests that ticks questing in fall did not come from the same cohort of larvae. Other studies have shown that ticks nymphs infected with *Borrelia* have higher energy levels, higher survival and a higher level of questing activity^40^. Over the summer, the changes in pathogen prevalence might be caused by a combination of differences in questing behaviour of infected ticks and by a lower proportion of questing ticks compared with those feeding on hosts. The high prevalence of pathogens in nymphs following peak rodent years, as found in our study, is consistent with the dynamics reported in endemic LD areas of the United States^12^.

During the last decades, the distribution of deer had increased in Europe due to changes in climate, land use and harvesting practices^14^. These changes in distribution have played a role in the emergence of LD. However, these changes in deer populations alone are not sufficient to explain the emergence of LD, as LD has emerged also in areas with a stable or declining population density of deer. Our study highlights that for vector-borne diseases in complex systems, drivers of emergence may differ across ecosystems and depend on the range of deer densities observed. The continuing emergence of LD and other vector-borne diseases in the Northern Hemisphere will require detailed epidemiological knowledge for successful forecasting at their northern limits.

## Methods

### Study areas—regional descriptions

The LD incidence data come from across the whole of Norway. We grouped into four regions based on biogeography; East (Østfold, Akershus, Oslo, Hedmark, Oppland and Buskerud Counties); South (Vestfold, Telemark, Aust-Agder and Vest-Agder Counties); West (Rogland, Hordaland, Sogn and Fjordane, and Møre and Romsdal Counties); and North (Sør-Trøndelag, Nord-Trøndelag, Nordland, Troms and Finnmark Counties). These four regions span contrasting ecosystems. The West region is separated from East by a mountain range, which is a major climatic division with resulting differences in vegetation. Further, topography differs markedly between West and East. The West region has substantial differences from East/South in terms of climate (temperate maritime climate in West, more continental climate in South/East), forest composition (more deciduous forest in West, compared with a mainly coniferous forest in the South and East) and in terms of the large mammal community. The West region is dominated by red deer, while the South and East regions by roe deer and moose (Supplementary Table 1). This differs in the North region of Norway, which is dominated by moose. Region South can be seen as an intermediate, closer to the East than to West in terms of mammal community and forest composition. The geology in the region West is dominated by Precambrian gneiss, while region South and East is dominated by basement but with region South having some areas with sedimentary rocks in the inner parts of the Oslo fjord. Vegetation in the South includes areas with nemoral forest along the southernmost coast, that is, deciduous forest trees such as oak (*Quercus* spp.) requiring warmer habitat, while the forest is in the boreonemoral and boreal zones further inland in the South region and in the East region^41^. Also, region South has a warmer, more humid climate and a much higher LD incidence than East (typically being a dry inland climate). Region North has a colder climate and a very low LD incidence.

### Study areas—field sites

In addition to the analysis of LD incidence across the whole of Norway, we conducted detailed field studies, which included a deer exclosure experiment, questing tick surveys and associated *B. burgdorferi* s.l. prevalence, and tick burdens on red deer ear samples. The purpose of these studies was to determine the mechanisms for how deer can affect LD hazard. The detailed field studies were conducted in the Sogn and Fjordane County and in the Møre and Romsdal/Sør-Trøndelag County located in the West region of Norway (Supplementary Fig. 2). The climate in these regions is oceanic (www.eklima.no). The precipitation and temperature generally declines from the coast to the inland and from the lowland to higher altitudes. The snow depth and duration of snow cover increases along the same gradients. The topography is highly variable rising from the fjords and valleys to mountain peaks reaching more than 1,500 m above sea level. The study areas are forested and situated in the south to middle boreal zone. Domestic sheep (*Ovis aries*) are abundant in some areas, but mainly graze in alpine habitats above the main tick population.

### Experimental design—deer exclosures

As part of a vegetation–deer study, 10 exclosures with 10 controls each measuring 20 m × 20 m (that is, 0.04 hectares) were established in 2008. These exclosures and controls were located in mature mixed pine-deciduous forests with a closed canopy cover in Tingvoll municipality, Møre and Romsdal County, Norway. Controls and exclosures were separated by 20–60 m to avoid fence-edge effects while minimizing vegetation differences. The placement of pair-wise control and manipulation was randomized at each site. The fence was a woven-wire mesh (2-m tall) nailed to stakes that had been sunk into the ground with a top wire at ∼2.2 m. With a net width of 15 cm, rodents and other small mammals were unrestricted to enter and exit the exclosures. The fences may behaviourally exclude medium-sized mammals to a certain extent, even though the mesh size was large. The flagging for ticks was performed in the middle of the exclosures and controls, in a plot measuring 2 m × 10 m. The flagging of all sites was performed within a short period of time during the main questing period in the spring (12–17 May 2011). The flagging of each treatment pair was typically initiated within 30–45 min of the previous pair to ensure as similar conditions as possible for each replicate.

### Tick load on deer

The tick load data derived from 49 adult red deer (≥2.5 years; 12 males, 37 females) marked with GPS-collars (Televilt/Followit, Stockholm, Sweden; Vectronic, Berlin, Germany) during the years 2008–2011 (ref. 42). Individual deer data are derived from one ear from each animal sampled at time of harvest. All tick instar stages were later counted. The analysis was only done on nymphs as they dominated the samples.

### Flagging of questing ticks in the landscape

We used the cloth lure (or flagging method) to gather data on the abundance of questing adult and nymphal ticks^43^. The sampling was performed in the spring (May/early June) and fall (August/early September) along 34 transects in Sogn og Fjordane (*n*=12,082 (nymphs), 2009–2014) and 37 transects in Møre and Romsdal/Sør-Trøndelag (*n*=11,216 (nymphs), 2011–2013, two missing values for 2011) in areas from coast to inland, from low to high elevation and with variable local densities of red deer. Each transect consisted of 12 survey plots with 20–50 m in between^43^. Each survey plot was flagged with a towel (50 cm × 100 cm) over a 20-m^2^ plot (∼10 m × 2 m). Ticks were counted and removed every 2 m after two drags on each side of the towel. In the field, ticks from each transect were pooled and killed using ethanol and then dried and stored with silica beads at −20 °C.

### DNA extraction and pathogen detection

We analysed the prevalence of *Borrelia* spp. for individual ticks using real-time PCR (*n*=5,876). Statistical analysis of variation in prevalence was done on nymphs only (Sogn and Fjordane: *n*=3,324, Møre and Romsdal: *n*=1,233). On the basis of our previous work^44^, we followed a protocol where DNA extractions were optimized for ticks by modifying the incubation step of the Qiagen DNeasy 96 Blood & Tissue kit. Ticks, 2 mm zirconium oxide beads, 40 μl of proteinase K solution and 4 μl Antifoam-A (Sigma) were incubated at 56 °C overnight followed by 5 min of bead homogenization at 30 cycles per second using a Qiagen TissueLyser II (Redwood City, USA). If the ticks were not broken, they were crushed using wooden toothpicks. The remaining 160 μl of proteinase K solution was added, and the homogenized mixture was incubated for 1 h at 56 °C. Centrifugation was performed before removing seals at any step in the protocol to prevent the cross-contamination of samples. The mixture was then transferred to the DNeasy plates. We used a real-time PCR assay on a Roche LightCycler 480 Real-Time PCR instrument to detect *B. burgdorferi* s.l. The amplifications were performed in a total reaction volume of 10 μl, including 0.7 μM for both the *B. burgdorferi* forward (5′-CGAGTCTTAAAAGGGCGATTTAGT-3′) and reverse (5′-GCTTCAGCCTGGCCATAAATAG-3′) primers and 0.175 μM of the *B. burgdorferi* specific probe (5′-AGATGTGGTAGACCCGAAGCCGAGTG-3′) in addition to 5 μl 2 × LightCycler Probes Mastermix (Roche, Mannheim, Germany), 32 μl miliQ H_2_O and 1 μl template DNA. The PCR programme was run as follows: pre-incubation at 95 °C for 600 s; denaturation at 95 °C for 10 s; and elongation at 60 °C for 60 s, repeated for 45 cycles. Positive controls of *B. burgdorferi* s.l. were used in addition to negative controls from the extraction. The results were scored as positive, negative or unknown (Supplementary Table 4).

### LD incidence

Statistics on the incidence of LD (borreliosis) in humans (The official MSIS statistics of Norway) is available from 1991 to 2012 (ref. 21). LD is a notifiable disease in Norway, but only including systemic infections (stages 2 and 3). As data from 1993 to 1994 include all cases of LD (not only stages 2 and 3), we excluded data from these years. We only used data for instances when municipality of the tick bite was certain.

### Deer density

Data on harvest statistics are available for all municipalities of Norway from Statistics Norway. For Møre and Romsdal County, we also have data from 17 local management units (higher spatial resolution) with harvest density of red deer varying from 0.16 to 4.65 per km^2^ (in 2010). As a proxy for deer population density, we used the number of harvested deer per km^2^ of deer habitat defined by the management that provides the basis for harvest quotas. This index has been tested thoroughly against independent abundance data and has been widely used in demographic studies of deer. See Supplementary Note 1 for further information regarding this measure.

There were often strong trends in population numbers of deer due to the strong age structure effects. These effects are often referred to as population momentum, and the correlation from one year to the next might be high (Fig. 2). Furthermore, due to delays in the tick life cycle, we might expect strong time lags and trends over the years. Hence, disentangling the temporal variation in deer density from overall temporal trends is challenging. For questing tick abundance, *Borrelia* spp. prevalence and tick load, we used deer density of the previous year (lag 1) because most quested larvae will become nymphs the following year. Owing to the short time series, these data will mainly measure the spatial contrasts in deer density. For longer time spans of LD incidence data, we present a final model separating the spatial and temporal component of deer density at the scale of the municipality. The spatial deer density component is the mean deer density of the municipality over the whole period. The temporal component is the deviation of log(deer density) a given year from log(spatial deer density) of the municipality, that is, the log ratio of deer density to spatial deer density.

### Rodent abundance index

In Norway, the genospecies *B. afzelii* is dominant in questing ticks (61.6% (ref. 32); 68.4% (ref. 33)), which suggests that small mammals are the most important reservoir hosts rather than birds (*B. garinii* 23.4% (ref. 32); 20.8% (ref. 33)). We retrieved the harvest statistics per county from Statistics Norway to estimate the number of red foxes (*V. vulpes*). The recruitment of foxes is known to follow rodent numbers (Supplementary Note 1). Hence, we used the number of red fox to test the rodent abundance index [log (*N*_*t*_/*N*_*t*-1_)] against independent data on nesting success of birds of prey known to follow the rodent cycle in one county (Supplementary Fig. 3).

### Biodiversity

Following Turney *et al.*^23^, we used distribution maps of mammal species known to be tick hosts, in total 18 species (Supplementary Note 2). We used mammal species richness at the scale of municipality.

### Environmental covariates

On the basis of a previous analysis of a subset of the tick questing data^43^, we calculated landscape variables such as distance to the coast, distance to the fjord, slope and elevation for all survey plots along the transects. These variables are known to affect the microclimate for ticks in these coastal areas. For the incidence of LD, we calculated several covariates on land use. On the basis of official Norwegian maps available in GIS, we calculated distance to the coast from the centre of each municipality. Likewise, we used Universal Transverse Mercator (UTM) coordinates to measure latitude. From Statistics Norway, we retrieved data on the proportion of forested areas in each municipality, the proportion of agricultural land and the areas used for human settlements, and data on the population size and proportion of people living in cities.

### Climate covariates

For the purpose of our research, broad climate covariates were the most relevant data to consider. In addition, the North Atlantic Oscillation (NAO) is known to affect the climate in Norway. We used principal component analysis-based data from Jim Hurrell at the National Center for Atmospheric Research (https://climatedataguide.ucar.edu/climate-data/hurrell-north-atlantic-oscillation-nao-index-pc-based) on seasonal NAO for December–January–February, March–April–May, June–July–August and September–October–November. Owing to the life cycle of ticks, we considered lags up to 2 years.

### Statistical analyses

We analysed data on the variation in questing tick abundances and LD incidence with negative binomial models^43^,^45^ with either ‘municipality' or ‘local management unit ID' and ‘transect' as random terms. We used the library glmmADMB in R × 64 versus 3.1.2 (http://www.r-project.org/). We tested whether using zero-inflated models were necessary to achieve a good fit. The tick load was analysed using negative binomial models in the library MASS (as no random terms were needed). The prevalence of *Borrelia* spp. was analysed using logistic regression in the library LME4 (random terms as above). We assessed emergence by fitting a continuous year term to the LD incidence data^46^. All models were checked for how well residuals fitted the prediction. To achieve a good fit, several variables were either log-transformed or square root-transformed to linearize relationship with the response variable. Our response was the number of patients with known systemic infection of LD and a known municipality of the tick bite. We used as an offset the (log) number of people (population) in a municipality so that we in effect are modelling on the LD incidences. We checked for both spatial (using previous year's presence/absence of LD in neighbouring municipalities) and temporal (LD incidence lagged by 1 year) autocorrelation structure. The model selection (mainly using AIC and checking for consistency with bayesian information criterion (BIC)) was performed for a wide range of environmental covariates in addition to the relevant deer population indices (a summary given in Table 2). Collinearity was assessed by calculating variance inflation factors (VIFs^47^), only variables providing VIFs<4 were kept in the same model (Supplementary Table 2). No patterns were observed from plotting residuals of the final model of LD incidence against all explanatory variables. The final model had no strong remaining spatial pattern in residuals. There was no strong autocorrelation (97.5% of autocorrelation function (lag 1)<0.5) when evaluating the residuals from 92 municipalities with 5 or more years with positive counts of LD. See Supplementary Note 3 for details on modelling and variables included in specific models.

### Data availability

LD incidence data derive from the «Norwegian Surveillance System for Communicable Diseases' (MSIS) and are available from the Norwegian Institute of Public Health (http://www.fhi.no/artikler/?id=93861). Data on human demography, land use, and host populations are available from Statistics Norway (https://www.ssb.no/statistikkbanken).

## Additional information

**How to cite this article:** Mysterud, A. *et al.* Contrasting emergence of Lyme disease across ecosystems. *Nat. Commun.* 7:11882 doi: 10.1038/ncomms11882 (2016).

## Supplementary Material

Supplementary Information Supplementary Figures 1-3, Supplementary Tables 1-4, Supplementary Notes 1-3 and Supplementary References

## Figures and Tables

**Table 1:** Analysis of Lyme disease incidence.

**Parameter**	**Estimate**	**S.E.**	***Z***	***P***
Intercept	−11.831	0.149	−79.4	<0.001
log(spatial deer density+0.1)	0.711	0.103	6.91	<0.001
Temporal deer density	0.085	0.039	2.20	0.028
Region (North versus West)	−1.174	0.305	−3.85	<0.001
Region (South versus West)	0.830	0.201	4.12	<0.001
Region (East versus West)	−0.959	0.239	−4.01	<0.001
Year	0.638	0.066	9.69	<0.001
Square root (distance to coast)	−1.225	0.137	−8.97	<0.001
Square root (proportion of residential settlement area)	−0.578	0.067	−8.57	<0.001
Spatial autocorrelation (lag 1)	0.276	0.083	3.33	<0.001
Latitude	−0.500	0.172	−2.90	0.004
NAO-JJA (lag 1)	0.197	0.033	5.95	<0.001
NAO-MAM (lag 2)	−0.125	0.026	−4.79	<0.001
NAO-DJF (lag 1)	0.131	0.026	4.98	<0.001
Year × region (North versus West)	0.170	0.152	1.12	0.262
Year × region (South versus West)	−0.331	0.073	−4.56	<0.001
Year × region (East versus West)	0.035	0.110	0.32	0.749

DJF, December–January–February; JJA, June–July–August; MAM, March–April–May; NAO, North Atlantic Oscillations.

Parameter estimates of the most parsimonious negative binomial model explaining the incidence of Lyme disease during 1991–2012 in Norway. All numeric variables were standardized. Baseline for region is West.

**Table 2:** Model selection of Lyme disease incidence.

	**ΔAIC**
*Excluding*	
Region	78.5
Spatial deer density	42.8
Temporal deer density	2.9
Year × region	25.3
Year × region+temporal deer density	52.1
Year	134.1
Square root (distance to coast)	76.3
Square root (proportion of residential settlement area)	53.6
Latitude	6.6
NAO-DJF (lag 1)	62.4
NAO-MAM (lag 2)	20.9
NAO-JJA (lag 1)	40.9
NAO-DJF (lag 1)+NAO-MAM (lag 2)+NAO-JJA (lag 1)	67.3
NAO (all)+temporal deer density+year × region	107.2
NAO (all)+year+temporal deer density	214.4
Spatial autocorrelation (lag 1)	9.3
All fixed effects (intercept and random effects only)	708.2
All fixed and random effects (intercept only)	2,259.6
	
*Replacing*	
Log (deer density) replacing spatial deer density+temporal deer density	7.1
Temporal deer density (lag 1) replacing temporal deer density	−0.8
	
*Adding*	
Temporal autocorrelation	−0.8
Proportion of people living in city	1.1
Proportion of agricultural area	−5.1
Proportion of forest	1.6
Mammal species richness	0.6
Rodent abundance index (lag 1)†	0.3

DJF, December–January–February; JJA, June–July–August; MAM, March–April–May; NAO, North Atlantic Oscillation index.

Difference in the AIC, DAIC, between the top-ranked (best) model in main Table 1 (AIC.6,031.2) and a model excluding, replacing or adding indicated explanatory variable*. Observation unit is number of LD cases in each municipality and year. There are in total 19 years and 416 municipalities with complete set of covariates giving 7,904 observation units over which 2,007 LD cases were found. Top-ranked model is the most parsimonious model with lowest AIC value or AIC not larger than 5 to other models. Parameters directly relevant for the testing of hypothesis were added if improving model fit and retained if significant. Spatial values are at scale of municipality, while temporal values are at scale of year lagged one (lag 1) or two (lag 2) years before to match life cycle of ticks.

^*^The top-ranked (best) model is the one parametrized in Table 1 and used as a starting point. We then challenge this model by either excluding those explanatory variables that are in the best model, adding those explanatory variables that are not in the best model, or replacing explanatory variables in the best model with related, correlated terms (that should not be in the same model).

^†^Compared with top-ranked model run on reduced data set (1 year lacking for this variable).

**Table 3:** Analysis of tick abundance in Møre og Romsdal County.

**Parameter**	**Estimate**	**S.E.**	***Z***	***P***	**ΔAIC**
Intercept	−0.284	0.1808	1.57	0.116	
Season (spring versus fall)	0.1124	0.0619	1.82	0.069	1.3
Elevation	−0.3930	0.1158	−3.39	<0.001	9.1
Distance to coast	−1.8587	0.3193	−5.82	<0.001	34.3
Year (2012 versus 2011)	0.0199	0.0743	0.27	0.789	131.0
Year (2013 versus 2011)	−0.8045	0.0786	−10.23	<0.001	
Slope	0.3107	0.0055	6.03	<0.001	35.0
log(spatial red deer density)	0.7778	0.2990	2.60	0.009	2.2
log(spatial red deer density)^2^	0.4409	0.2038	2.16	0.031	2.3
					
*Excluding*					
All variables					224.2
All variables and random effects					1604.5
Zero-inflation					9.4

Parameter estimates from analysis of abundance of questing ticks in spring and autumn with a zero-inflated negative binomial model (AIC=10,020.8) and ‘transect' as random terms for the West region, Møre and Romsdal county, Norway. Sample size is 11,216 nymphal ticks from 37 transects for 2 seasons each of 3 years. Each transect consists of 12 survey plots, and observation unit is survey plot for a given transect, season and year (in total *n*=2,614). Baseline values are year 2011 and season Fall. ‘Distance to coast' was entered as the mean for a local management unit. ‘Slope', ‘elevation' and ‘distance to coast' are scaled to mean zero and variance one. The ΔAIC refers to the effect of removing the variable in the given row from the given model and at bottom excluding terms.

**Table 4:** Analysis of tick abundance in Sogn og Fjordane County.

**Parameter**	**Estimate**	**S.E.**	***Z***	***P***	**ΔAIC**
Intercept	2.6225	0.2912	9.005	<0.001	
Season (spring versus fall)	0.3099	0.0567	5.470	<0.001	27.3
Year (2010 versus 2009)	−0.0456	0.1028	−0.444	0.657	173.5
Year (2011 versus 2009)	−0.5834	0.0855	−6.827	<0.001	
Year (2012 versus 2009)	−0.3151	0.1060	−2.973	0.003	
Year (2013 versus 2009)	−0.9644	0.1202	−8.024	<0.001	
Year (2014 versus 2009)	−1.1810	0.1067	0.270	<0.001	
Elevation	0.0109	0.0784	0.139	0.889	27.5
(Elevation)^2^	−0.3281	0.0590	−5.565	0.000	29.5
bs(distance to coast)1	−8.3404	1.2578	−6.631	0.000	94.7
bs(distance to coast)2	−1.7015	1.5149	−1.123	0.261	
bs(distance to coast)3	−5.2523	0.8259	−6.360	0.000	
Slope	0.4002	0.0375	10.672	<0.001	118.6
Log(red deer density)	−0.0328	0.0818	−0.401	0.689	13.7
Log(red deer density)^2^	0.1894	0.0448	4.223	<0.001	15.7
					
*Excluding*					
All variables					536.7
All variables and random effects					2573.0
					
Replacing					
Year category replaced by year numeric					33.2
Log (red deer density) (lag 1) replaced by log (spatial deer density)					10.9
					
Adding					
Zero-inflation					1.6

Parameter estimates from analysis of abundance of questing ticks with a negative binomial model (AIC=13,317.1) with ‘transect' as random terms for the West region, Sogn & Fjordane county, Norway. Sample size is 12,082 nymphal ticks from 34 transects for 2 seasons each of 6 years. Each transect consists of 12 survey plots, and observation unit is survey plot for a given transect, season and year (in total *n*=4,419). Continuous variables are scaled to mean zero and variance one. Baseline values are year ‘2009' and season ‘fall'. ‘Red deer density': red deer density for a municipality. ‘Distance to coast' was modelled as third-order polynomial using a cubic spline function (bs). The ΔAIC refers to the effect of removing the variable in the given row, and at bottom if excluding, replacing and adding variables from the best model.

**Table 5:** Analysis of tick load on red deer.

**Parameter**	**Estimate**	**S.E.**	***Z***	***P***	**ΔAIC**
Intercept	12.3947	2.0524	6.039	<0.001	
Red deer density	1.1918	0.3367	3.539	<0.001	9.9
Julian date	−0.0300	0.0055	−5.460	<0.001	21.4
Carcass mass	−0.0396	0.0124	−3.195	0.001	4.7
Elevational difference between summer and winter range	−0.0032	0.0010	−3.300	<0.001	7.2
Excluding all variables (intercept only)					35.4

Parameter estimates and test statistics for the analysis of tick load on ears of red deer (*n*=49) in the West region, Norway. Model AIC=204.1. The ΔAIC refers to the effect of removing a given variable from the model and at bottom excluding all variables.

**Table 6:** Analysis of pathogen prevalence.

**Parameter**	**Estimate**	**S.E.**	***Z***	***P***
S&F—May—using year				
Intercept	−1.6877	0.5237	−3.223	0.001
Year (2010 versus 2009)	1.0016	0.4210	2.379	0.017
Year (2011 versus 2009)	0.9045	0.4074	2.220	0.026
Year (2012 versus 2009)	0.6993	0.4296	1.628	0.104
Year (2013 versus 2009)	−1.2884	0.6415	−2.009	0.045
Year (2014 versus 2009)	0.7980	0.4289	1.860	0.063
Red deer density (lag 1)	−0.6147	0.1931	−3.182	0.001
				
S&F—May—using Rodent abundance index				
Intercept	−1.3702	0.3149	−4.352	0.000
Rodent abundance index	2.4879	1.0851	2.293	0.022
Red deer density (lag 1)	−0.4456	0.1779	−2.504	0.012
				
S&F—Aug—using year				
Intercept	−2.7669	0.5531	−5.003	0.000
Year (2011 versus 2009)	−0.1564	0.2660	−0.588	0.557
Year (2012 versus 2009)	−0.5680	0.2984	−1.903	0.057
Year (2013 versus 2009)	−0.1203	0.2762	−0.435	0.663
Year (2014 versus 2009)	0.1582	0.2890	0.547	0.584
Red deer density (lag 1)	0.4129	0.2734	1.510	0.131
				
S&F—Aug—using Rodent abundance index				
Intercept	−2.9885	0.4695	−6.365	0.000
Rodent abundance index	−1.2321	1.5119	−0.815	0.415
Red deer density (lag 1)	0.4711	0.2559	1.841	0.066
				
M&R				
Intercept	−1.8763	0.2546	−7.371	<0.001
Year (2013 versus 2011)	0.5620	0.1789	3.141	0.002
Red deer density (lag 1)	−0.5077	0.2122	−2.392	0.017

M&R, Møre and Romsdal; S&F, Sogn and Fjordane.

Parameter estimates and test statistics for the analysis of prevalence of *Borrelia burgdorferi* sensu lato in nymphal *Ixodes ricinus* ticks in two counties in the West region, Norway. Analyses are separate for county Sogn and Fjordane (S&F) in May and August (due to lacking data in 2010 for August) and for county Møre and Romsdal (M&R). In M&R, there was no effect of ‘season' (ΔAIC=1.60) or interaction ‘season × year' (ΔAIC=2.0) if added to the model. For S&F, due to longer time series, we were able to run models using either year or the rodent abundance index. Random terms were ‘municipality' (*n*=9) for S&F and ‘local management unit' (*n*=21) for M&R.
