# Supplementary material for: Contrasting emergence of Lyme disease across ecosystems
Source: Nat Commun. 2016 Jun 16;7:11882. doi: 10.1038/ncomms11882 (PMC4912636; doi:10.1038/ncomms11882)
Supplement: Supplementary Information — Supplementary Figures 1-3, Supplementary Tables 1-4, Supplementary Notes 1-3 and Supplementary References [file ncomms11882-s1.pdf]

Supplementary Figures

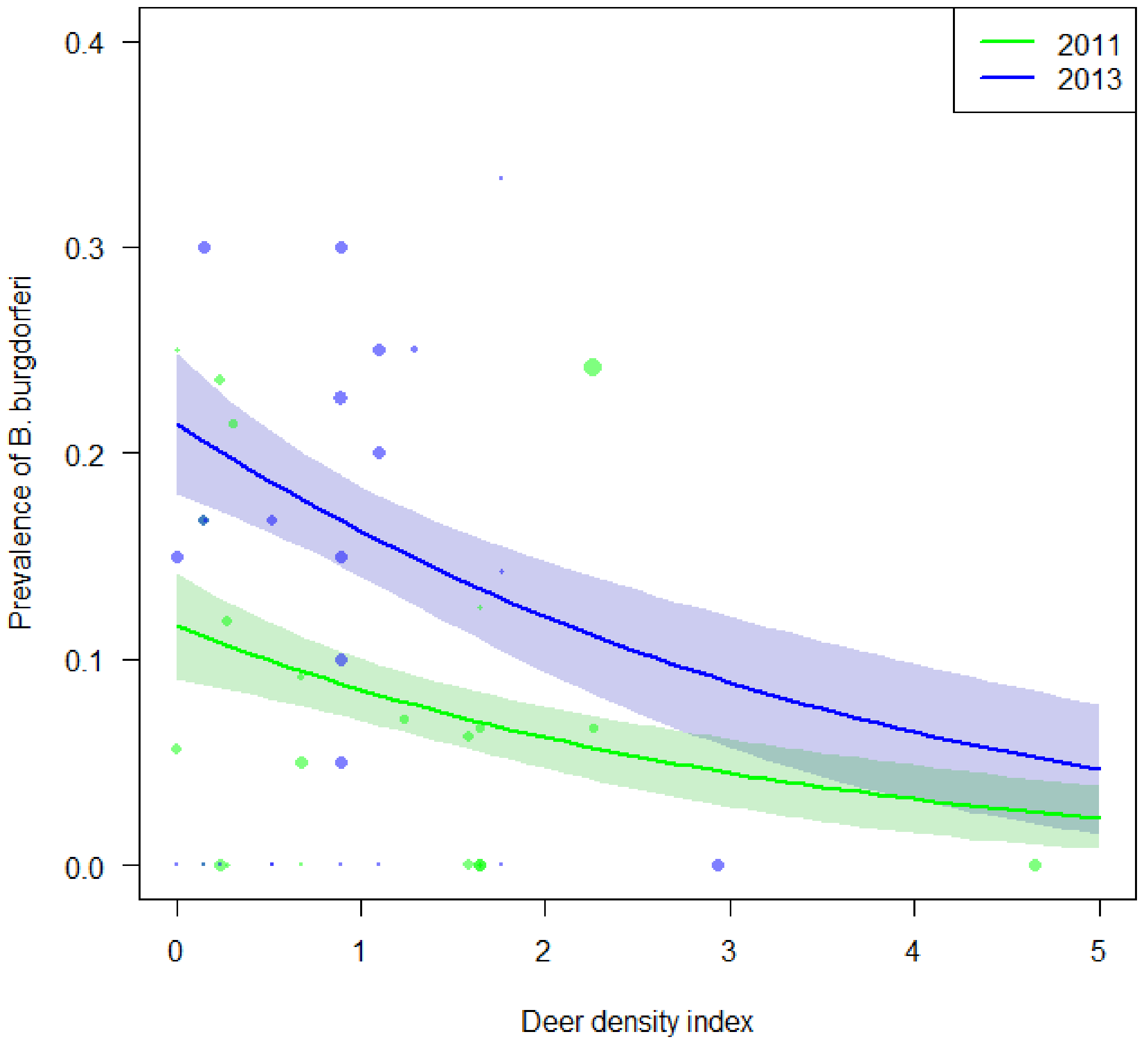

Supplementary Fig. 1. The relationship between the prevalence of *Borrelia* spp. in ticks and deer density index in county Møre & Romsdal (M&R), region “west” of Norway. Data points are proportional to (sqrt) sample size.

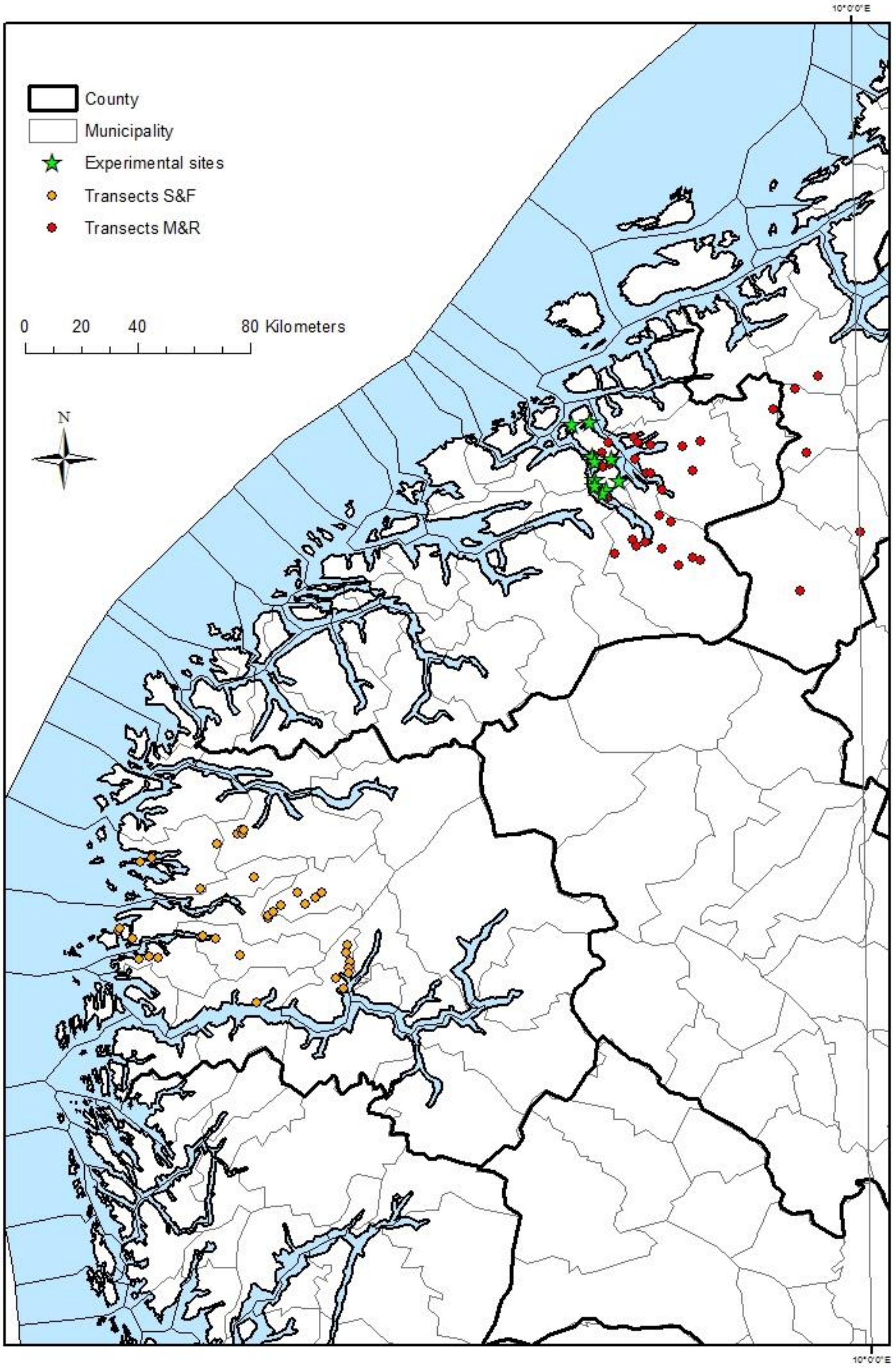

Supplementary Fig. 2. The position of transects and experimental sites for measurements of abundances of questing ticks and pathogen dilution in the "west" region, counties Sogn & Fjordane (S&F) and Møre & Romsdal/Sør-Trøndelag (M&R), Norway.

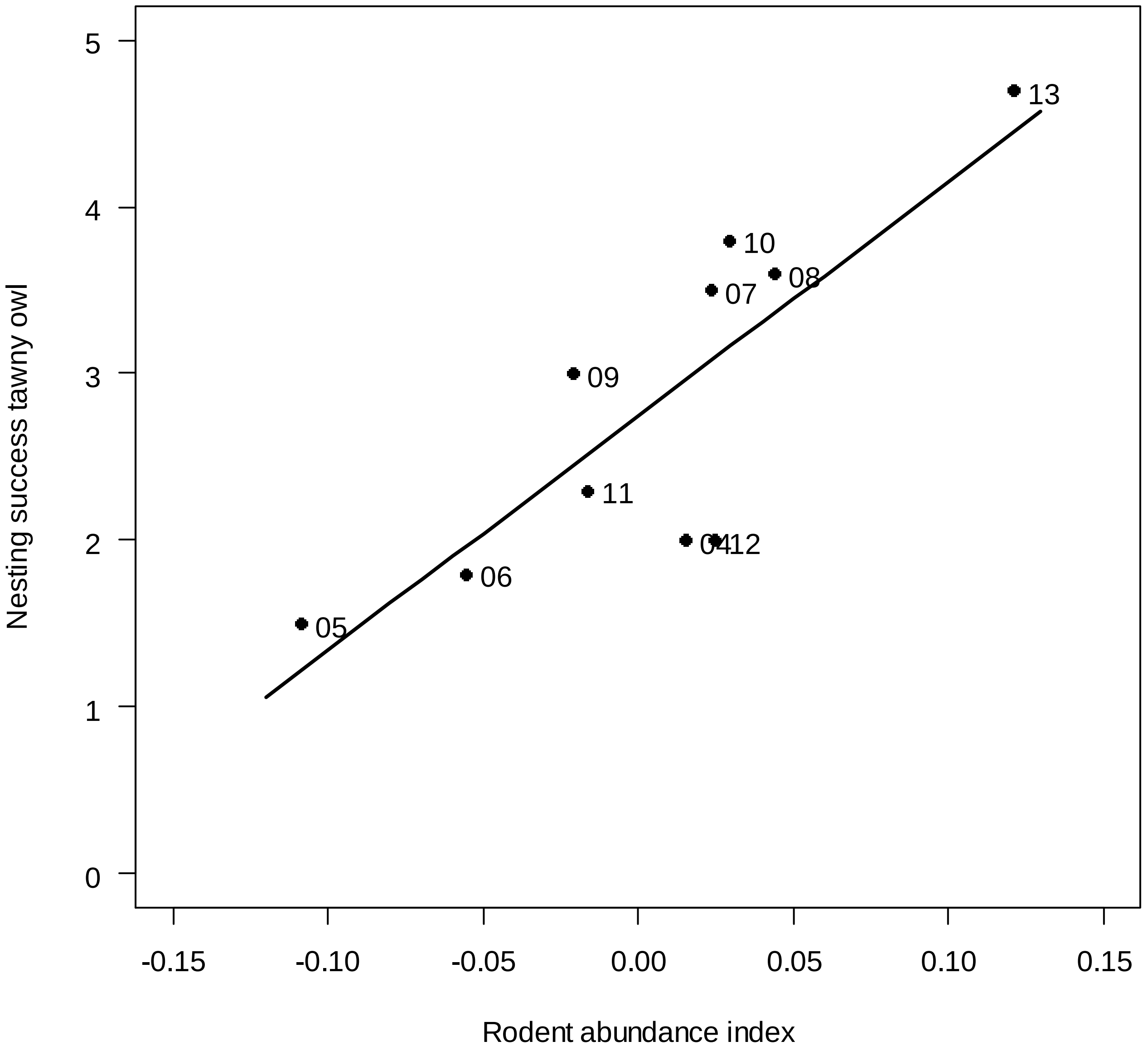

Supplementary Fig. 3. The relationship between rodent abundance index (log (N_t/N_{t-1}); harvest of red fox) from Møre & Romsdal county and nesting success of tawny owl in Rindal municipality, Møre & Romsdal county, Norway for the period 2004-2013 (denoted 04-13).

Supplementary Table 1. An overview of range of deer harvest densities (10, 50 and 90% percentiles) in the four regions of Norway, 1991-2011. The category deer is the sum of red deer, roe deer and moose. It is the number of harvested deer per km^2 of suitable deer habitat (mainly forest, removing alpine areas) as approved by deer management authorities.

	1991			2001			2011		
	10%	50%	90%	10%	50%	90%	10%	50%	90%
**Deer**									
West	0.16	0.43	0.78	0.34	0.86	1.67	0.59	1.39	2.37
East	0.23	0.67	1.81	0.26	0.66	1.83	0.30	0.66	1.49
South	0.22	1.00	1.72	0.32	0.91	1.92	0.29	0.78	1.84
North	0	0.13	0.62	0.01	0.17	0.73	0.07	0.27	1.16
**Red deer**									
West	0	0.32	0.67	0.02	0.72	1.52	0.18	1.13	2.12
East	0	0	0.008	0	0	0.02	0	0.004	0.07
South	0	0	0.004	0	0.004	0.02	0	0.04	0.08
North	0	0	0.04	0	0	0.06	0	0	0.12
**Roe deer**									
West	0	0	0.33	0	0	0.51	0	0	0.77
East	0.05	0.30	1.55	0.03	0.21	1.44	0.02	0.21	1.02
South	0.07	0.40	1.29	0.04	0.36	1.57	0.07	0.47	1.65
North	0	0	0.25	0	0	0.23	0	0	0.48
**Moose**									
West	0	0	0.02	0	0	0.03	0	0	0.04
East	0.12	0.29	0.57	0.16	0.37	0.57	0.19	0.33	0.60
South	0.12	0.36	0.78	0.15	0.36	0.59	0.09	0.20	0.35
North	0	0.08	0.33	0	0.13	0.45	0.03	0.21	0.61

Supplementary Table 2 | An overview of pairwise Pearson correlations between spatial variables for the model of Lyme disease incidence in the whole of Norway. We used variance inflation factors (VIF) to asses which variables are collinear, using a cutoff of 4 to remove collinear variables[1].

	Distance to the coast (sqrt)	Latitude	Prop. of agriculatural area (sqrt)	Prop. of forest area	Prop. of residential settlement area (sqrt)	Prop. of people living in city (sqrt)	Mammal species richness	Spaital deer density (log)
Distance to coast (sqrt)	1	-0.22	-0.14	0.32	-0.28	-0.07	0.43	-0.21
Latitude		1	-0.40	-0.43	-0.37	-0.18	-0.75	-0.66
Prop. of agricultural area (sqrt)			1	0.12	0.61	0.28	0.33	0.59
Prop. of forest area				1	0.19	0.14	0.65	0.34
Prop. of residential settlement area area (sqrt)					1	0.48	0.28	0.56
Prop. of people living in city (sqrt)						1	0.21	0.26
Mammal species richness							1	0.46
Deer density (log)								1

Supplementary Table 3 | An overview of empirical work linking the parameters questing tick abundance or tick load on hosts ("density"), prevalence of *Borrelia burgdorferi* sensu lato in ticks ("prevalence"), tick disease hazard (density of infected nymphs; "hazard") and Lyme disease incidence ("incidence") or TBE incidence in humans to population density of deer. No = No effect; Neg/Pos = a negative/positive relationship to deer density. Ref. refers to numbering in Supplementary References.

Parameter	Scale (replication)	Deer density range (index)	Species of deer	State/country	Conclusion	Ref.
North America						
Density, prevalence, hazard	Temporal (1993-2005)	Not given (deer observed per hour hunted, deer browse surveys)	White-tailed deer	New York	No	[2]
Density	Spatial contrast (1986)	Not given (direct observation)	White-tailed deer	New York	Pos	[3]
Density, incidence	Temporal (2002-2005)	24.3 vs. 45.6 deer km^{-2}	White-tailed deer	New Jersey	No	[4]
Density	Spatial (2 high vs. 2 low)	Not given (deer browsing, deer pellet groups)	White-tailed deer	New Jersey	No	[5]
Density	Spatial (9 transects at each of 3 sites)	Not given, activity rather than density (distance to deer trails, bait stations)	White-tailed deer	New Jersey	No	[6]
Density	Spatial (2.7 km transects, 3 sites)	Not given, activity rather than density (pellet groups)	White-tailed deer	Maine	No	[7]
Density	Temporal (before/after deer removal from island)	0 vs. 37 deer km^{-2}	White-tailed deer	Maine	Pos	[8]

Density	Spatial (8 sites of 5.2 km^2)	<7.1 - 55 deer km^{-2} (pellet group counts)	White-tailed deer	Maine	Pos	9
Density	Temporal (1983-86 vs. 1987-91)	Not given, reduction of density by harvest	White-tailed deer	Massachusetts	Pos (neg. for adult ticks, likely short-term effect of deer removal)	10
Density, incidence	Temporal (1995-2007)	0-10 vs. 39.8-54.5 deer km^{-2}	White-tailed deer	Connecticut	Pos	11,12
Density	Temporal (1992-2001)	10-17 vs. >90 deer km^{-2} (aerial surveys, spotlight counts)	White-tailed deer	Connecticut	Pos	13
Incidence	Temporal, before treatment (1992-1998), after treatment (1999-2006)	Deer density not accounted for, but treatment of deer with acaricide using four-poster stands	White-tailed deer	Connecticut	Pos	14
Incidence	Temporal (30 years), spatial contrast (4 states)	Not given; ~2x increase in index (antlered deer harvest by hunting license sales, raw antlered-deer harvest data)	White-tailed deer	Minnesota, Wisconsin, Virginia, Pennsylvania	No (variable)	15
Density, prevalence, hazard	Spatial contrast (12 sites)	Not given (pellet group counts)	White-tailed deer	Ontario, Canada	Pos (no effect on prevalence)	16
Europe						
Density	Spatial contrast (fence)	No vs. high (pellet group counts)	Fallow deer	Ireland	Pos	17
Prevalence	Spatial	No vs. high	Fallow	Ireland	Neg	17

	contrast (fence)	(pellet group counts)	deer			
Density	Spatial contrast (55 sites)	Not given (pellet group counts)	Roe deer, red deer	Scotland	Pos	[18,19,20]
Density, prevalence	Spatial contrast (25 sites)	Not given (pellet group counts)	Roe deer, red deer	Scotland	Pos	[21]
Prevalence	Spatial contrast (5 islands)	Deer harvest density; <1 vs. >3 deer harvested km^{-2} (~<4 vs. >15 deer km^{-2})	Roe deer, red deer, moose	Norway	Neg	[22]
Prevalence	Spatial contrast (9 municipalities)	Deer harvest density; 0.75-2.4 harvested deer km^{-2} (~3.5-12.0 deer km^{-2})	Red deer	Norway	Neg	[23], this study
Incidence	Spatial (416 municipalities) and temporal (1991-2011)	Deer harvest density	Red deer, roe deer, moose	Norway	Pos	this study
Density	Spatial contrast (9 forest departments)	2.4-9.1 deer km^{-2} (Distance sampling) Deer harvest density; 3.5-6.7 harvested deer km^{-2}	Roe deer	Germany	Pos	[24]
Prevalence	Spatial contrast (2 areas)	<20 vs. >100 deer km^{-2}	Roe deer, red deer, fallow deer	France	No	[25]
Incidence of tick-borne encephalities	Spatial contrast (17 areas), temporal (1992-2006)	1.84-4.34 roe deer km^{-2}	Roe deer	Italy	Pos	[26]
		0.28-0.75 red deer km^{-2}	red deer		No	[26]

Density, incidence of tick-borne encephalities	Spatial contrast (6 areas)	Not given (pellet group counts)	Roe deer	Italy	Pos (humped)	[27,28]
Incidence of tick-borne encephalities	Temporal (1970-2008), spatial contrast (204 units)	0-8.39 red deer km^{-2}	Red deer	Slovenia, Europe	Pos	[29]
		Average 8.79 roe deer km^{-2}	Roe deer		No	[29]

Note: Tick species are *Ixodes scapularis* in USA and *Ixodes ricinus* in Europe. The *Borrelia burgdorferi* sensu lato genospecies causing Lyme disease are *Borrelia burgdorferi* sensu stricto in USA and *Borrelia afzelii*, *Borrelia garinii* and to a lesser extent *Borrelia burgdorferi* sensu stricto in Europe. Deer species are white-tailed deer (*Odocoileus virginianus*) in the USA and roe deer (*Capreolus capreolus*), red deer (*Cervus elaphus*), fallow deer (*Dama dama*) and moose (*Alces alces*) in Europe. We have not included all exclosure experiments, as debates regarding size of exclosures have been done previously[18,59,60].

Supplementary Table 4 | An overview of the samples being positive of *Borrelia burgdorferi* sensu lato in *Ixodes ricinus* nymphs and the total sample size (n) collected with flagging in May and August 2009-2014 in counties Sogn & Fjordane and 2011 and 2013 in Møre & Romsdal, region "west" of Norway. These data were used to calculate prevalence (positive/n).

		positive	n	prevalence (%)
Sogn & Fjordane				
2009	May	7	125	5.60
	Aug	33	270	12.22
2010	May	46	342	13.45
	Aug	-	-	-
2011	May	82	669	12.26
	Aug	33	308	10.71
2012	May	39	310	12.58
	Aug	21	299	7.02
2013	May	4	201	1.99
	Aug	31	306	10.13
2014	May	37	289	12.80
	Aug	27	204	13.24
Sum/Mean	May	215	1936	11.11
	Aug	145	1387	10.45
Møre & Romsdal				
2011	May	30	384	7.81
	Aug	28	379	7.39
2013	May	61	384	15.89
	Aug	67	324	20.68
Sum/Mean	May	91	768	11.85
	Aug	95	703	13.51

Supplementary Notes

Supplementary Note 1. Using harvest statistics as measure of population abundance. Harvest statistics in general may provide biased estimates of population abundances, as they also may reflect hunter effort[30] and harvest quotas[31]. The population indices used here have been thoroughly tested against independent data to assess their reliability as indices of population trends. We here briefly review the evidence for using them and their limitations.

A. Deer density index. As a deer population density index, we used the number of harvested deer per km^2 of deer habitat as defined by management providing the basis for harvest quotas[32,33]. This so-called counting area forms the main basis for setting quotas rather than number of deer per see. We retrieved counting area for roe deer, red deer and moose from Statistics Norway, and we used the largest available figure for each municipality. In most cases this will be the same, but in some areas with only one species this is important to control for.

This deer density index have been tested against independent abundance data and has been used widely in demographic studies[32,34], showing clear links to deer performance such as body mass[32], age at first reproduction and timing of ovulation[34], suggesting it reflects density relative to resource levels and is mainly caused by management differences[32]. For red deer, the harvest density correlated with population density estimated from cohort analysis[35]. Similarly for moose, the harvest density of moose correlated with population density estimated from cohort analysis both within and between regions[36]. There might be 1-2 year lag between the true population size and the harvest size due to the quota system (for moose[31,37]), but the most recent analysis suggests a good correlation between the harvest density of moose and population dynamics[36]. For roe deer, harvest density index is used in analysis of population dynamics and regarded a very good proxy for population size[38,39].

For 2008, there are no official statistics available for moose and red deer, and we interpolated between 2007 and 2009. The correlation between red deer density one year with the next was high (e.g. for red deer: 2010-2011: r=0.98; 2011-2012: r=0.97). For roe deer, historical data before 2008 was retrieved from researchers having gathered these directly from the municipalities[38]. If missing values, typically occurring in areas with few roe deer, we used lambda derived from the log harvest (Yt/Yt-1) at the county scale to predict the number at the lacking year at the scale of municipality.

B. Rodent abundance index. Red fox (*Vulpes vulpes*) numbers are known to follow rodent numbers, and such data have been used to analyse rodent cycles in Norway[40] and elsewhere[41]. We used the log (N_t/N_{t-1}) to account for potential bias in trends of variable hunter effort over time. We had access to a time series (2004-2013) of number of fledged/hatched young for the tawny owl (*Strix aluco*) from Rindal municipality, Møre & Romsdal (Lars Løfaldli, unpubl. data). Nesting success of the tawny owl is linked to rodent cycles (R.A. Ims, pers. comm.), and there was a close linear fit between our rodent abundance index and the nesting success of the tawny owl, in line with previous studies confirming the red fox harvest can be used as a proxy for rodent abundance (Supplementary Figure 3). Note, however, that the rodent abundance index is calculated at a county scale, i.e., at a much coarser scale than municipality for which we have other data.

Supplementary Note 2. Biodiversity.

We used mammal host species richness as a measure of biodiversity, similar to Turney *et al*.[42] for North America. Note that *Borrelia afzelii* (small mammal reservoir) rather than *Borrelia garinii* (bird reservoir) dominate in Norway[43-46], suggesting small mammals rather than birds are the more important for LD in Norway. According to the most authorative reference for Norway, there are 15 mammals that are known hosts to *Ixodes ricinus* if excluding humans and 6 domestic species[47]. From other studies, we have added to this list roe deer and moose[48,49]. We also discussed the list with the author of the early review (a well-known tick expert, Reidar Mehl) to make sure we had not missed species being described later.

We used the most updated account of mammal distribution of Norway[50], based on advice from a mammal expert from the Norwegian Zoological Society (Kjell Isaksen). We checked for consistency with another account[51], and there were very few differences in species distributions between. For cervids (roe deer, red deer and moose), we used distribution data from Statistics Norway, as these species are all harvested very soon if they appear in a new area[52]. For each species, we recorded whether the species was present in a given municipality based on the maps. For each municipality, the number of species recorded was then used as a measure of biodiversity.

Supplementary Note 3. A detailed overview of model specifications.

We here give some detailed background on the modelling approaches used. Most of this is regarding LD incidence being the by far most comprehensive analyses due to its spatial and temporal extent. But the main approach of how to achieve a good model fit, model selection and presentation of results are similar to all analyses.

Model fit and appropriate residuals. An overall important aim is to achieve an appropriate model fit; most importantly that there are no remaining patterns in the residuals. This was achieved by accounting for potential dependency between observations by the aid of a random term ("municipality") and terms for spatial and temporal autocorrelation. Our data had frequent zero observations. This was modelled by negative binomial models with low mean (μ) and overdispersion. We tested whether using zero-inflation were necessary to achieve a good fit. Further, we linearized relationships by aid of appropriate transformations such as "log" (ln-transformed) or "square root". To achieve appropriate fit of more strongly non-linear relationships, we also tried polynomial terms or cubic splines. All numeric variables was standardized to facilitate comparison of effect sizes (i.e., scaled to mean zero and variance one). Due to the life history of ticks, we lagged relevant covariates 1 or 2 years, denoted as "[lag1]" and "[lag 2]".

Model selection. The model selection (mainly using AIC and checking for consistency with BIC) was performed for a wide range of environmental covariates in addition to the relevant deer population indices. Collinearity was assessed by calculating variance inflation factors (VIF's[53]), only variables providing VIF's < 4 were kept in the same model. We used both forward and backward selection as well as testing biologically guided removal of several variables at the same time to ensure that sequence of adding variables was not an issue. To avoid overfitting, we used a fairly high ΔAIC value for guiding inclusions into the final model. When we had a well-fitting model, we present more formally the results in table 2 of how removal, replacing or adding variables affect model performance in terms of ΔAIC. Due to fewer covariates in other models, the best models and the effect of removing terms are presented together in tables 3-6. The ΔAIC presents the effect of removing the variable in the given row.

Table presentation of results from best model. Results are presented in term of estimates from the best models. These estimates are so-called treatment contrasts, i.e. relative to a baseline. For example, our baseline is the region "west" (table 1). The estimate for region

“south” (0.830) is thus how much LD incidence in “south” differs from that in the region “west” (log scale). This also implies that the estimate for the main effect of “Year” (0.638) is for region “west”, and that the negative estimate for “Year * Region (south vs. west)” (-0.331) is relative to the baseline for “west”. That is, the estimated effect for “Year” in region “south” is (0.638-0.331=0.307). Hence, the estimated trend for “Year” is positive (0.307) also in the region “south”, but significantly lower than in region “west” (0.638). Notice that estimated effects are given on log scale (due to the log link of the negative binomial model).

Figure presentation of results from best model. In figures 1C, 2C and 2D, the lines are predictions from the model for year- and region-specific average conditions of all other covariates. The residuals are based on the actual set of variables, and may hence by chance be slightly above or below the fitted lines, if the values for a given combination of covariates differ from the average conditions used for drawing the line. Negative binomial models have a large scatter (dependent on the μ relative to the dispersion parameter). To enhance presentation, the residuals are therefore binned over a range (of x-values). The same approach of presenting results is used in figure 3, with lines being predictions for average conditions and points being residuals (not binned due to lower sample size).

Table 1 and 2: Analysis of LD incidence

The raw data are number of cases of systemic stages of Lyme disease with a known origin of the tick bite. We are interested in analysing the LD incidence rather than the number of LD cases, and hence need to account for human population size. Since “cases per capita” are not normally distributed, incidence can better be modelled with the negative binomial (NB) distribution and an offset variable (log population size) (ref. [1], page 241).

The following variables were tested for, but not all included in the final model in table 1.

Terms to account for potential dependency in data

- “Municipality”. A random term to account for repeated observations from the same area.
- “Spatial autocorrelation”. This was accounted for by including for each year and municipality an index (1-0) of “Previous year’s presence of LD in neighbouring municipalities”.
- “Temporal autocorrelation”. This was “Previous year’s incidence of LD cases in the municipality”.

Descriptive terms

- "Year". A continuous term for year to assess temporal changes in LD incidence. This is used to formally test for an emergence, defined here as an increase in LD incidence over years. If this terms remain significant after adding a driver (such as deer density), it is evidence this driver alone cannot explain the emergence.
- "Region". A 4 level categorical variable (west, east, south, north) separating the main nature-geographic regions of Norway with different deer population developments (see study area section).

Land use variables

- "Proportion of residential settlement area". The proportion of the area in the municipality with human settlements.
- "Proportion of agricultural area". The proportion of the area in the municipality with agriculture.
- "Proportion of forest". The proportion of the area in the municipality with forest.
- "Proportion of people living in city". The proportion of the human population size within each municipality living in cities rather than in more rural environments.

Deer density indexes. If not specified, deer refers to sum of red deer, roe deer and moose. See Supplementary Note 1. The best model included "Spatial deer density" and "Temporal deer density".

- "Deer density". Number of deer harvested per km^2 in a given year in a given municipality.
- "Spatial deer density". The mean number of deer harvested per km^2 in a given municipality over the entire time period.
- "Temporal deer density". The residual between log number of deer harvested in a given year minus the log mean number of deer harvested per km^2 in a given municipality over the entire time period. (i.e., the log ratio of "Deer density" to "Spatial deer density") for each year).

Indices of other mammals

- "Mammal species richness". The number of mammalian tick host species recorded in the municipality. See Supplementary Note 2.
- "Rodent abundance index". An annual value for the rodent population size. See Supplementary Note 1.

Climate proxy variables (spatial). These variables are used to control for local climate variation in space for which there are no direct estimates of climate. Analysis of tick abundance shows a strong effect of "distance to coast" in these highly coastal regions[54] and also with latitude[55]. These indices hence predict tick population abundance in Norway, but not necessarily in other regions.

- "Distance to coast". Distance from coast to centre of a given municipality.
- "Latitude". The latitude in UTM at the centre of a given municipality.

Climate variables (temporal): the North Atlantic Oscillation (NAO) index is well known to have a major influence on climate in Norway, and it is used extensively in ecological research[56]. The benefit of using such indexes rather than local measurements is that it accounts for climate over a broader scale, cfr. refs. [57,58].

- "NAO- JJA". The NAO index for months June-July-August.
- "NAO- MAM": The NAO index for months March-April-May.
- "NAO- DJF": The NAO index for months December-January-February.

Table 3: Analysis of tick questing abundance in MR

The response variable was the "number of questing nymphal ticks" a negative binomial model was used. Data derive from flagging surveys in county Møre og Romsdal (MR) at the scale of local management unit (smaller than municipality), in Norway.

- "local management unit". A random term to account for possible dependency between measurements from the same unit.
- "Year". Year as a categorical term. This was years 2009-14 for the SF area and 2011-13 for the MR area.
- "Season". This was a categorical variable with two levels ("spring", "fall").
- "Spatial red deer density". The mean number of red deer harvested per km^2 in a given local management unit over the entire time period.

Climate proxy variables (spatial):

- "Elevation". The elevation in m above sea level for each survey plot.
- "Slope". The slope at each survey plot.
- "Distance to coast". This is the mean distance to fjord within each "local management unit", as the mean values gave a better fit than distance to fjord at each survey plot and was hence retained.

Table 4: Analysis of tick questing abundance in SF

The response variable was the "number of questing nymphal ticks" a negative binomial model was used. Data derive from flagging surveys in county Sogn og Fjordane (SF) at the scale of municipalities in Norway.

- "municipality". A random term to account for possible spatial dependency.
- "Year". Year as a categorical term. This was years 2009-14 for the SF area and 2011-13 for the MR area.
- "Season". This was a categorical variable with two levels ("spring", "fall").
- "Red deer density". Number of red deer harvested per km^2 in a given year in a given municipality.

Climate proxy variables (spatial):

- "Elevation". The elevation in m above sea level for each survey plot.
- "Slope". The slope at each survey plot.
- "Distance to coast". This was distance to nearest fjord in m from each survey plot.

Table 5: Tick load analysis

The response variable was the "number of nymphal ticks" on ears from GPS-marked red deer and a negative binomial model was used. The negative binomial model included the following fixed effects:

- "Red deer density". Number of red deer harvested per km^2 in a given year in a given municipality.
- "Julian date". A continuous variable counting days from January 1st, as number of ticks on deer decline from September onwards.
- "Carcass mass". Eviscerated body mass (kg) of red deer being ~55% of live mass.
- "Elevational difference between summer and winter range". The difference in elevation of the seasonal centre of the home range during winter and summer.

Table 6: Analysis of prevalence of *Borrelia burgdorferi* sensu lato

The response variable was the prevalence of *Borrelia burgdorferi* sensu lato and hence a binomial model was used. Analysis was done separately for each season and the two counties SF and MR. Note that "Year" (as categorical) and "Rodent index" was never entered in the same model. This is because the "Rodent index" is an annual index and there would be no residual variation if first having year (as categorical) in the model.

Step 1 using "Year" as categorical:

- "municipality" (for SF) or "local management unit" (for MR). A random term to account for possible spatial dependency.
- "Year". Year as a categorical term. This was years 2009-14 for the SF area and 2011 and 13 for the MR area.
- "Red deer density". Number of red deer harvested per km^2 in a given year in a given municipality.

Step 2 using "Rodent index":

- "municipality" (for SF) or "local management unit" (for MR). A random term to account for possible spatial dependency.
- "Rodent index". An annual value for the rodent population size. See Supplementary Note 1.
- "Red deer density". Number of red deer harvested per km^2 in a given year in a given municipality.

Supplementary References

1. Zuur, I. Leno, E. N. Walker, N. Saveliev, A. A. & Smith, G. M. *Mixed effects models and extensions in ecology with R.* (Springer, New York, 2009).
2. Ostfeld, R. S., Canham, C. D., Oggenfuss, K., Winchcombe, R. J. & Keesing, F. Climate, deer, rodents, and acorns as determinants of variation in Lyme-disease risk. *Plos Biol* **4**, 1058-1068 (2006).
3. Wilson, M. L., Ducey, A. M., Litwin, T. S., Gavin, T. A. & Spielman, A. Microgeographic distribution of immature *Ixodes dammini* ticks correlated with that of deer. *Med Vet Entomol* **4**, 151-159 (1990).
4. Jordan, R. A., Schulze, T. L. & Jahn, M. B. Effects of reduced deer density on the abundance of *Ixodes scapulatis* (Acari: ixodidae) and Lyme disease incidence in a northern New Jersey endemic area. *J Med Entomol* **44**, 752-757 (2007).
5. Jordan, R. A. & Schulze, T. L. Deer browsing and the distribution of *Ixodes scapularis* (Acari: Ixodidae) in Central New Jersey forests. *Environ Entomol* **34**, 801-806 (2005).
6. Schulze, T. L., Jordan, R. A. & Hung, R. W. Potential effects of animal activity on the spatial distribution of *Ixodes scapularis* and *Amblyomma americanum* (Acari: Ixodidae). *Environ Entomol* **30**, 568-577 (2001).

7. Lubelczyk, C. B., Elias, S. P., Rand, P. W., Holman, M. S., Lacombe, E. H. & Smith, R. P. Habitat associations of *Ixodes scapularis* (Acari: Ixodidae) in Maine. *Environ Entomol* **33**, 900-906 (2004).
8. Rand, P. W., Lubelczyk, C., Holman, M. S., Lacombe, E. H. & Smith, R. P. Abundance of *Ixodes scapularis* (Acari: Ixodidae) after the complete removal of deer from an isolated offshore island, endemic for Lyme disease. *J Med Entomol* **41**, 779-784 (2004).
9. Rand, P. W., Lubelczyk, C., Lavigne, G. R., et al. Deer density and the abundance of *Ixodes scapularis* (Acari: Ixodidae). *J Med Entomol* **40**, 179-184 (2003).
10. Deblinger, R. D., Wilson, M. L., Rimmer, D. W. & Spielman, A. Reduced abundance of immature *Ixodes dammini* (Acari: Ixodidae) following incremental removal of deer. *J Med Entomol* **30**, 144-150 (1993).
11. Kilpatrick, H. J., LaBonte, A. M. & Stafford, K. C. The relationship between deer density, tick abundance, and human cases of Lyme disease in a residential community. *J Med Entomol* **51**, 777-784 (2014).
12. Kilpatrick, H. J. & LaBonte, A. M. Deer hunting in a residential community: the community's perspective. *Wildl Soc Bull* **31**, 340-348 (2003).
13. Stafford, K. C., DeNicola, A. J. & Kilpatrick, H. J. Reduced abundances of *Ixodes scapularis* (Acari: Ixodidae) and the tick parasitoid *Ixodiphagus hookeri* (Hymenoptera: Encyrtidae) with reduction of white-tailed deer. *J Med Entomol* **40**, 642-652 (2003).
14. Garnett, J. M., Connally, N. P., Stafford, K. C. & Cartter, M. L. Evaluation of deer-targeted interventions on Lyme disease incidence in Connecticut. *Public Health Reports* **126**, 446-454 (2011).
15. Levi, T., Kilpatrick, A. M., Mangel, M. & Wilmers, C. C. Deer, predators, and the emergence of Lyme disease. *Proc Natl Acad Sci USA* **109**, 10942-10947 (2012).
16. Werden, L., Barker, I. K., Bowman, J., et al. Geography, deer, and host biodiversity shape the pattern of Lyme disease emergence in the Thousand Islands Archipelago of Ontario, Canada. *Plos One* **9**, e85640(2014).
17. Gray, J. S., Kahl, O., Janetzki, C. & Stein, J. Studies on the ecology of Lyme disease in a deer forest in County Galway, Ireland. *J Med Entomol* **29**, 915-920 (1992).
18. Gilbert, L., Maffey, G. L., Ramsay, S. L. & Hester, A. J. The effect of deer management on the abundance of *Ixodes ricinus* in Scotland. *Ecol Appl* **22**, 658-667 (2012).
19. Gilbert, L. Altitudinal patterns of tick and host abundance: a potential role for climate change in regulating tick-borne diseases? *Oecologia* **162**, 217-225 (2010).

20. Ruiz-Fons, F. & Gilbert, L. The role of deer as vehicles to move ticks, *Ixodes ricinus*, between contrasting habitats. *Int J Para* **40**, 1013-1020 (2010).
21. James, M. C., Bowman, A. S., Forbes, K. J., Lewis, F., McLeod, J. E. & Gilbert, L. Environmental determinants of *Ixodes ricinus* ticks and the incidence of *Borrelia burgdorferi* sensu lato, the agent of Lyme borreliosis, in Scotland. *Parasitology* **140**, 237-246 (2013).
22. Rosef, O., Paulauskas, A. & Radzijevskaja, J. Prevalence of *Borrelia burgdorferi* sensu lato and *Anaplasma phagocytophilum* in questing *Ixodes ricinus* ticks in relation to the density of wild cervids. *Acta Vet Scand* **51**, 47(2009).
23. Mysterud, A., Easterday, W. R., Qviller, L., Viljugrein, H. & Ytrehus, B. Spatial and seasonal variation in prevalence of *Anaplasma phagocytophilum* and *Borrelia burgdorferi* in *Ixodes ricinus* ticks in Norway. *Parasite Vector* **6**, 187(2013).
24. Vor, T., Kiffner, C., Hagedorn, P., Niedrig, M. & Rühe, F. Tick burden on European roe deer (*Capreolus capreolus*). *Exp Appl Acarol* **51**, 405-517 (2010).
25. Pichon, B., Mousson, L., Figureau, C., Rodhain, F. & Perez-Eid, C. Density of deer in relation to the prevalence of *Borrelia burgdorferi* sl in *Ixodes ricinus* nymphs in Rambouillet forest, France. *Exp Appl Acarol* **23**, 267-275 (1999).
26. Rizzoli, A., Hauffe, H. C., Tagliapietra, V., Neteler, M. & Rosà, R. Forest structure and roe deer abundance predict tick-borne encephalitis risk in Italy. *Plos One* **4**, e4336(2007).
27. Bolzoni, L., Rosà, R., Cagnacci, F. & Rizzoli, A. Effect of deer density on tick infestation of rodents and the hazard of tick-borne encephalitis. II: Population and infection models. *Int J Para* **42**, 373-381 (2012).
28. Cagnacci, F., Bolzoni, L., Rosa, R., et al. Effects of deer density on tick infestation of rodents and the hazard of tick-borne encephalitis. I: Empirical assessment. *Int J Para* **42**, 365-372 (2012).
29. Knap, N. & Avsic-Zupanc, T. Correlation of TBE incidence with red deer and roe deer abundance in Slovenia. *Plos One* **8**, e66380(2013).
30. Willebrand, T., Hörnell-Willebrand, M. & Asmyhr, L. Willow grouse bag size is more sensitive to variation in hunter effort than to variation in willow grouse density. *Oikos* **120**, 1667-1673 (2011).
31. Fryxell, J. M., Packer, C., McCann, K., Solberg, E. J. & Sæther, B.-E. Resource management cycles and the sustainability of harvested wildlife populations. *Science* **328**, 903-906 (2010).

32. Mysterud, A., Yoccoz, N. G., Stenseth, N. C. & Langvatn, R. The effects of age, sex and density on body weight of Norwegian red deer: evidence of density-dependent senescence. *Proc R Soc Lond Ser B* **268**, 911-919 (2001).

33. Mysterud, A., Stenseth, N. C., Yoccoz, N. G., Langvatn, R. & Steinheim, G. Nonlinear effects of large-scale climatic variability on wild and domestic herbivores. *Nature* **410**, 1096-1099 (2001).

34. Langvatn, R., Mysterud, A., Stenseth, N. C. & Yoccoz, N. G. Timing and synchrony of ovulation in red deer constrained by short northern summers. *Am Nat* **163**, 763-772 (2004).

35. Mysterud, A., Meisingset, E. L., Veiberg, V., et al. Monitoring population size of red deer: an evaluation of two types of census data from Norway. *Wildl Biol* **13**, 285-298 (2007).

36. Ueno, M., Solberg, E. J., Iijima, H., Rolandsen, C. M. & Gangsei, L. E. Performance of hunting statistics as spatiotemporal density indices of moose (*Alces alces*) in Norway. *Ecosphere* **5**, 13(2014).

37. Solberg, E. J., Sæther, B.-E., Strand, O. & Loison, A. Dynamics of a harvested moose population in a variable environment. *J Anim Ecol* **68**, 186-204 (1999).

38. Grøtan, V., Sæther, B.-E., Engen, S., et al. Climate causes large-scale spatial synchrony in population fluctuations of a temperate herbivore. *Ecol* **86**, 1472-1482 (2005).

39. Mysterud, A. & Østbye, E. The effect of climate and density on individual and population growth of roe deer *Capreolus capreolus* at northern latitudes - the Lier valley, Norway. *Wildl Biol* **12**, 321-329 (2006).

40. Henden, J.-A., Ims, R. A. & Yoccoz, N. G. Nonstationary spatio-temporal small rodent dynamics: evidence from long-term Norwegian fox bounty data. *J Anim Ecol* **78**, 636-645 (2009).

41. Jacquot, M., Coeurdassier, M., Couval, G., et al. Using long-term monitoring of red fox populations to assess changes in rodent control practices. *J Appl Ecol* **50**, 1406-1414 (2013).

42. Turney, S., Gonzalez, A. & Millien, V. The negative relationship between mammal host diversity and Lyme disease incidence strengthens through time. *Ecol* **95**, 3244-3250 (2014).

43. Franke, J., Hildebrandt, A. & Dorn, W. Exploring gaps in our knowledge on Lyme borreliosis spirochaetes-updates on complex heterogeneity, ecology, and pathogenicity. *Ticks Tick Borne Dis* **4**, 11-25 (2013).

44. Kjelland, V., Stuen, S., Skarpaas, T. & Slettan, A. Prevalence and genotypes of *Borrelia burgdorferi* sensu lato infection in *Ixodes ricinus* ticks in southern Norway. *Scand J Infect Dis* **42**, 579-585 (2010).
45. Tveten, A. K. Prevalence of *Borrelia burgdorferi* sensu stricto, *Borrelia afzelii, Borrelia garinii,* and *Borrelia valaisiana* in *Ixodes ricinus* ticks from the northwest of Norway. *Scand J Infect Dis* **45**, 681-687 (2013).
46. Tveten, A. K. Exploring diversity among Norwegian *Borrelia* strains originating from *Ixodes ricinus* ticks. *Int J Microbiol* **Article ID 397143**, (2014).
47. Mehl, R. The distribution and host relations of Norwegian ticks (Acari, Ixodides). *Fauna norvegica Series B* **30**, 46-51 (1983).
48. Handeland, K., Qviller, L., Vikøren, T., Viljugrein, H., Lillehaug, A. & Davidson, R. K. *Ixodes ricinus* infestation in free-ranging cervids in Norway - a study based upon ear examinations of hunted animals. *Vet Parasitol* **195**, 142-149 (2013).
49. Kjelland, V., Ytrehus, B., Stuen, S., Skarpaas, T. & Slettan, A. Prevalence of *Borrelia burgdorferi* in *Ixodes ricinus* ticks collected from moose (*Alces alces*) and roe deer (*Capreolus capreolus*) in southern Norway. *Ticks Tick Borne Dis* **2**, 99-103 (2011).
50. Hagström, T. & Hagström, E. *Mammals of Norden (In Norwegian)* (Gyldendal, Oslo, 2011).
51. Bjärvall, A. & Ullström, S. *Mammals. All European species in text and picture.* (Cappelen, Oslo, 1997).
52. Milner, J. M., Bonenfant, C., Mysterud, A., Gaillard, J.-M., Csányi, S. & Stenseth, N. C. Temporal and spatial development of red deer harvesting in Europe - biological and cultural factors. *J Appl Ecol* **43**, 721-734 (2006).
53. James, G. Witten, D. Hastie, T. & Tibshirani, R. *An introduction to statistical learning with applications in R.* (Springer, New York, 2013).
54. Qviller, L., Risnes-Olsen, N., Bærum, K. M., et al. Landscape level variation in tick abundance relative to seasonal migration pattern of red deer. *Plos One* **8**, e71299(2013).
55. Jore, S., Viljugrein, H., Hofshagen, M., et al. Multi-source analysis reveals latitudinal and altitudinal shifts in range of *Ixodes ricinus* at its northern distribution limit. *Parasite Vector* **4**, 84(2011).
56. Stenseth, N. C., Ottersen, G., Hurrell, J. W., et al. Studying climate effects on ecology through the use of climate indices: the North Atlantic Oscillation, El Niño Southern Oscillation and beyond. *Proc R Soc Lond Ser B* **270**, 2087-2096 (2003).

57. Hallett, T. B., Coulson, T., Pilkington, J. G., Clutton-Brock, T. H., Pemberton, J. M. & Grenfell, B. Why large-scale climate indices seem to predict ecological processes better than local weather. *Nature* **430**, 71-75 (2004).
58. Stenseth, N. C. & Mysterud, A. Weather packages: Finding the right scale and composition for climate in ecology. *J Anim Ecol* **74**, 1195-1198 (2005).
59. Pugliese, A. & Rosa, R. Effect of host populations on the intensity of ticks and the prevalence of tick-borne pathogens: how to interpret the results of deer experiments. *Parasitology* **135**, 1531-1544 (2008).
60. Perkins, S. E., Cattadori, I. M., Tagliapietra, V., Rizzoli, A. P. & Hudson, P. J. Localized deer absence leads to tick amplification. *Ecol* **87**, 1981-1986 (2006).
